# Benchmarking adult CT-dose levels to regional and national references using a dose-tracking software: a multicentre experience

**DOI:** 10.1007/s13244-017-0570-5

**Published:** 2017-09-07

**Authors:** Lotte Pyfferoen, Tom H. Mulkens, Federica Zanca, Timo De Bondt, Paul M. Parizel, Jan W. Casselman

**Affiliations:** 1 Department of Radiology, AZ Sint-Jan Brugge-Oostende AV, Ruddershove 10, 8000 Brugge, Belgium; 2 Department of Radiology, H. Hart Hospital, Mechelsestraat 24, 2500 Lier, Belgium; 3 GE Healthcare, DoseWatch, Rue de la Minière 283, 78530 Buc, France; 4 Imaging and Pathology Department, KU Leuven, Herestraat 49, 3000 Leuven, Belgium; 5 Department of Radiology, Antwerp University Hospital and University of Antwerp, Wilrijkstraat 10, 2650 Antwerp, Belgium

**Keywords:** Adult, Computed tomography, Radiation dosage, Multicentre study, Radiation monitoring

## Abstract

**Objectives:**

To benchmark CT-dose data for standard adult CT studies to regional and national reference levels using a dose-tracking system.

**Methods:**

Data from five CT systems from three hospitals were collected over a 1- to 2.5-year period (2012–2014), using the same type of dose management system. Inclusion criteria were adult patients and standard CT-head, CT-abdomen-pelvis, CT-thorax, CT-lumbar spine, CT-pulmonary embolism, CT-cervical spine and CT-thorax-abdomen studies, with one helical scan. Volumetric CT-dose index (CTDI_vol_), dose length product (DLP) and scan length from 31,709 scans were analysed statistically.

**Results:**

After dose optimisation CTDI_vol_ and DLP values were below the national diagnostic reference levels (DRLs) for all CT studies and for all systems investigated. Mostly no significant differences were found between CTDI_vol_ and DLP levels (*p* values ≥ 0.01) of CT studies performed on different scanners within the same hospital. Significant dose differences (*p* values < 0.01) were instead observed among hospitals for comparable CT studies. Dose level range and scan length differences for similar CT studies were revealed.

**Conclusions:**

Dose-tracking systems help to reduce CT-dose levels below national DRLs. However, dose and protocol data comparison between and within hospitals has the potential to further reduce variability in dose data of standard adult CT studies.

***Key Points*:**

• *Retrospective three-centre study on dose levels of standard adult CT procedures.*

• *Dose-tracking systems help hospitals to stay below national dose reference levels.*

• *Dose-tracking systems help to align CT dose levels between scanners within hospitals.*

• *Benchmarking shows CT dose level variability for similar examinations in different hospitals.*

• *Differences in dose level range/scan length for similar CT studies are revealed.*

**Electronic supplementary material:**

The online version of this article (10.1007/s13244-017-0570-5) contains supplementary material, which is available to authorized users.

## Introduction

The availability of equal healthcare for all patients across Europe and the reduction of risks in patient care are key ambitions of the European Commission and World Health Organisation [1, 2]. As computed tomography (CT) technology evolves, many new applications have emerged, leading to high numbers of CT scans performed. Today CT is a major contributor to patient radiation exposure.

Radiation doses used to perform similar CT studies of diagnostic quality should remain within a relatively narrow range. However, national and multinational surveys indicate that this is not the case; large variability in dose levels exists [1–4]. Therefore, the ICRP introduced the concept of the ‘diagnostic reference level’ (DRL), with the objective of providing a reference level for the radiation dose for standard radiographic and CT examinations [5]. In 2013 the European Union published the results of the Dose Datamed II project, including the DRLs of 26 European countries [6]. As equipment, procedures and protocols may vary among different facilities and areas, national DRLs based on the 75th percentile (P75) are being used. Further dose optimisation below this value is possible [7]. Determination of dose reference levels and dose optimisation in general might be facilitated by using dose-tracking software, as it allows collection of a large amount of data. Recent literature studies [8, 9] already showed the potential of automated collection of a large amount of data, allowing comparison between local volumetric CT-dose index (CTDI_vol_) and dose length product (DLP) values and the national DRLs. In addition, dose-tracking software systems facilitate awareness of radiographers and radiologists about the radiation doses delivered to patients.

DRLs should be used to identify hospitals that are routinely using higher or lower CT dose levels and to generate triggers to optimise radiation doses according to the ALARA (As Low As Reasonably Achievable) principles. Such efforts should result in a decrease of DRLs. However, when the DRLs of different European countries are compared [10–21] from 1999 to 2014, one can conclude that the values did not change substantially over time. There are several reasons for this. At first, the gradual replacement of single-detector row CT scanners with multi-detector row CT scanners starting from 1998 required larger doses because of the nature of their geometry. Besides, patient sizes have increased [22]. Another factor is the time needed to implement legislation and to organise and analyse nationwide surveys. Also, new dose optimisation CT technologies (‘tube current modulation’ in 2004–2005 [23] and ‘iterative reconstruction techniques’ [24] in 2010) are probably not yet reflected in the DRL data of all countries.

The hypothesis of this multicentre study is that a dose management system, with a large amount of data collection, will demonstrate significant variation in dose levels for similar adult CT examinations performed in different institutions in a comparable region of Europe. Using a dose-tracking system also showed that in one hospital CT dose levels were modified to levels below the national DRLs and that it is easy to compare and align CT dose levels between scanners within one hospital. The resulting inter-hospital benchmarking will change the used protocol parameters and CT dose levels and could eventually influence the regional and national DRLs.

## Materials and methods

### Patient data collection

To monitor early effects of using dose-tracking software on CT dose levels, CT data were collected and anonymised in three different hospitals from the start of the integration of dose-monitoring software, with a minimum collection period of 1 year (January 2012, January 2013 and July 2013 respectively until June 2014). The included hospitals were a university hospital (A), a large-size city hospital (B) and a smaller regional hospital (C), located in the same geographical region. All CT examinations of patients under 18 years were excluded from the study because the paediatric patient data had already been used in another study. The use of routinely collected CT data was approved by the Institutional Review Board (Medical Ethics Committee) of the three hospitals, and the requirement for informed consent of this retrospective study was waived.

The five CT systems used in this study are listed in Table 1. All scanners were equipped with automatic tube current modulation and iterative reconstruction software, ASiR (Adaptive Statistical iterative Reconstruction), on GE CT systems (General Electric Healthcare, Milwaukee, WI, USA) and IRIS (Iterative Reconstruction in Image Space) or SAFIRE (Sinogram AFfirmed Iterative REconstruction) on Siemens CT systems (Siemens Healthcare, Erlangen, Germany).

**Table 1:** CT systems used in the three hospitals

Hospital	CT name	Manufacturer	System	Year of installation	Detector rows
A	CT 1	GE	Lightspeed VCT	2009	64
A	CT 2	GE	Lightspeed VCT	2008	64
B	CT 3	GE	Discovery HD750	2009	64
C	CT 4	Siemens	Emotion	2012	16
C	CT 5	Siemens	Somatom definition AS+	2012	128

The same real-time dose-tracking system, DoseWatch version 1.3 (GE Healthcare, Milwaukee, WI, USA), was used to collect and retrieve the following data directly from the CT and Picture Archiving and Communication System (PACS) devices: (1) age and sex, (2) protocol parameters, (3) dosimetry-related data including the scan length, CTDI_vol_ (32-cm body phantom and 16-cm head phantom) and DLP and (4) total number of irradiation series (without localiser and bolus tracking). A revision of the quality control tests, performed yearly in the three hospitals, was assessed to ensure that the displayed dosimetric values were correct. The use of contrast agents and their effects on radiation dose were not evaluated as this was out of scope of this study. Procedure-related information such as the clinical indication was collected manually from the Radiology Information System (RIS) and PACS of each hospital and/or from the medical records of the patients.

Only the data of the most frequently performed ‘CT regions’ (CTs of different anatomical regions) were included. These were the data requested by our national regulatory body and for which national DRLs were available: CT-head, CT-abdomen-pelvis, CT-thorax, CT-lumbar spine, CT-pulmonary embolism, CT-cervical spine and CT-thorax-abdomen. Different CT-protocol names existing for the same CT region (e.g. CT-stroke, CT-head trauma) were grouped under the reference CT region (e.g. CT-head) and had to be associated with the most frequent clinical indications of that CT region; see Table 2.

**Table 2:** Anatomical “CT regions” and associated most frequent clinical indications

CT protocol name	Most frequent clinical indications
CT-head	Cerebrovascular accident
	Head trauma
	Headache
	Acute neurological deficit
	Dementia
CT-abdomen-pelvis	Acute abdomen
	Abdominal or gastrointestinal cancer
	Gallbladder or pancreatic disease
CT-thorax	Lung cancer
	Cough
	Lung infection
	Pleural disease
	Interstitial lung disease
	Trauma
CT-pulmonary embolism	Detection or exclusion of pulmonary embolism
CT-lumbar spine	Low back pain with sciatica
	Trauma
CT-thorax-abdomen	Staging and follow-up of tumours
	Inflammatory pathology
	Unexplained weight loss
CT-cervical spine	Neck pain with brachial plexopathy/neuropathy
	Trauma

Only CT studies consisting of one helical series per CT region were included. This implied exclusion of for example multiphase liver or renal examinations and CT-guided biopsies. On the series level, examinations in which the CT region did not match the ‘CT clinical indications’ were selectively eliminated. Also, series were ranked by scan length, and the longest and shortest scan length series were verified case by case and excluded when necessary (e.g. thoracolumbar spine scanned but registered as a ‘lumbar spine’ CT study).

For dose optimisation purposes, protocol and reconstruction parameters of CT-head and CT-abdomen-pelvis examinations were retrieved. A team consisting of a CT radiologist, a local DoseWatch coordinator and a CT application specialist of the vendor agreed upon the parameters to adapt. For hospital B the noise index of abdomen-pelvis examinations was changed from 35.42 to 38.64, resulting in lower effective mAs values, and for CT-head examinations the minimal mA was changed from 100 mA to 80 mA, resulting in lower effective mAs values. For CT-head examinations in hospital A it was noticed that the two identical scanners had a large difference in their noise indices. Protocols were harmonised according to the settings of the scanner with the largest noise index. The effect of changing the parameters on dose levels was monitored for another four months. Image quality was evaluated subjectively in the hospitals and the resulting images were considered of diagnostic quality by both the lead CT radiologists (one in each hospital with > 20 years of experience) and the radiologists with organ subspecialty (one or two per hospital with > 10 years of experience).

### Patient data analysis

The cleaned data were evaluated per CT region, age and sex. The median CTDI_vol_ and median DLP were calculated and compared to the third survey round of national DRLs, which were based on data acquired in the period of October 2012 – September 2013 [21]. Data management was performed using Excel version 2010 (Microsoft Corp., Redmond, WA, USA) and data were analysed statistically using Graphpad Prism version 6 (Graphpad software Inc., La Jolla, CA, USA). For the non-Gaussian distributed data, non-parametric Kruskal-Wallis test with post-hoc Dunn testing was used; *p* values < 0.01 were considered statistically significant. Outliers were detected using Tukey rules (outside 1.5× the interquartile range).

## Results

Together the CT-head, CT-abdomen-pelvis, CT-thorax, CT-pulmonary embolism, CT-lumbar spine, CT-thorax-abdomen and CT-cervical spine studies accounted for 34.9% (12,175 out of 34,934), 70.2% (11,172 out of 15,914) and 59.5% (7813 out of 13,124), of all adult CT studies performed in hospital A, B and C respectively. Of the 31,709 CT studies included, the most frequently performed scans were: CT-head (27.3%), CT-abdomen-pelvis (18.8%) and CT-thorax (17.0%); see Table 3. The age of the included patients ranged from 18 years to at least 95 years for all CT regions with a maximum of 101 years for a CT-head examination. The overall male/female ratio for all CT examinations was 1.0 with a clear male dominance for CT-thorax (1.40) and CT-thorax-abdomen (1.32), a slight male dominance for CT-pulmonary embolism (1.19) and a slight female dominance for all the other CT-regions (CT-head: 0.95, CT-abdomen-pelvis: 0.91, CT-lumbar spine: 0.83, CT-cervical spine: 0.78).

**Table 3:** Number of included CT examinations per CT system

	CT1	CT2	CT3	CT4	CT5	All CTs
CT-head	640	2208	3485	1419	912	8664 (27.3%)
CT-abdomen-pelvis	552	1984	1903	762	747	5948 (18.8%)
CT-thorax	612	1796	1909	557	527	5401 (17.0%)
CT-pulmonary embolism	612	2191	860	330	0	3993 (12.6%)
CT-lumbar spine	0	632	1369	1319	452	3772 (11.9%)
CT-thorax-abdomen	127	634	1041	0	204	2006 (6.3%)
CT-cervical spine	169	653	530	474	99	1925 (6.1%)
Total	2712	10,098	11,097	4861	2941	31,709 (100%)

**Fig. 1:**
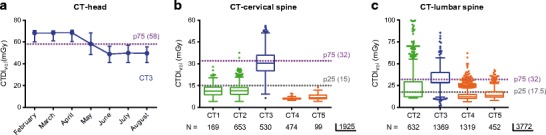
Using dose-tracking systems results in and detects CTDI_vol_ values below the national DRLs. **a** Graph showing the median CTDI_vol_ and interquartile range per month (period February 2013–August 2013) of CT-head examinations in hospital B with the national P75 DRL as benchmark. **b** Box plot of the CTDI_vol_-median value, interquartile range, total range and outliers for all CT-cervical spine examinations on the five CT systems with the national P75 and P25 DRLs as benchmark. **c** Box plot of the CTDI_vol_-median value, interquartile range, total range and outliers for all CT-lumbar spine examinations on four CT systems with the national P75 and P25 DRLs as benchmark

**Fig. 2:**
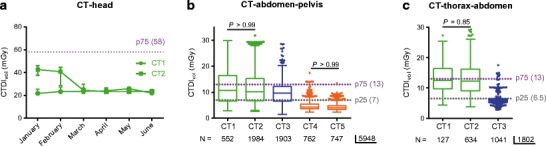
Effect of using dose-tracking systems on differences in CTDI_vol_ levels between scanners within one hospital. **a** Graph showing the median CTDI_vol_ and interquartile range per month (period January 2013–June 2013) of CT-head examinations for the two CT systems in hospital A with the national P75 DRL as benchmark. **b** Box plot of the CTDI_vol_-median value, interquartile range, total range and outliers for all CT-abdomen-pelvis examinations on the five CT systems with the national P75 and P25 DRLs as benchmark, demonstrating median CTDI_vol_ levels between both CT systems in hospital A and between both CT systems in hospital C are not different. **c** Box plot of the CTDI_vol_-median value, interquartile range, total range and outliers for all CT-thorax-abdomen examinations on three CT systems with the national P75 and P25 DRLs as benchmark, demonstrating median CTDI_vol_ levels between both CT systems in hospital A and between both CT systems in hospital C are not different. Kruskal-Wallis test with post-hoc Dunn test. Adjusted *p* values not shown on the graph are below 0.01

**Fig. 3:**
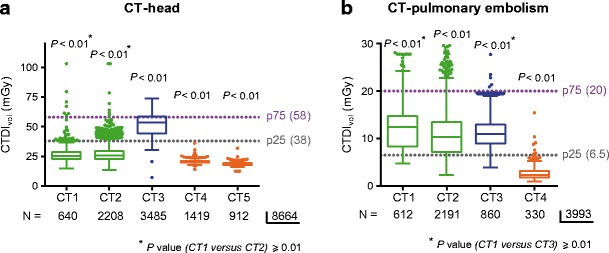
Detection of differences in dose levels used in different hospitals for similar anatomical regions by dose-tracking systems illustrating that different hospitals are using the highest dose depending on the anatomical region involved. **a** Box plot of the CTDI_vol_-median value, interquartile range, total range and outliers for all CT-head examinations on the five CT systems with the national P75 and P25 DRLs as benchmark, showing hospital B has the highest CTDI_vol_. *p* values (all < 0.01) are the result of comparing the mean rank of each CT system with the mean rank of each other CT system. **b** Box plot of the CTDI_vol_-median value, interquartile range, total range and outliers for all CT-pulmonary embolism examinations on four CT systems with the national P75 and P25 DRLs as benchmark, showing the CTDI_vol_ is not statistically different (*p* value of CT1 versus CT3 ≥ 0.01) between CT system 1 in hospital A and the CT system in hospital B. All other *P* values (< 0.01) are the result of comparing the mean rank of each CT system with the mean rank of each other CT system. Kruskal-Wallis test with post-hoc Dunn test

**Fig. 4:**
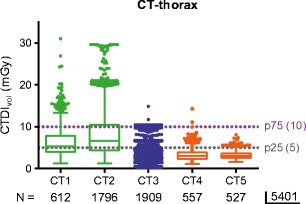
Detection of differences in the range of dose levels for CT-thorax studies in the participating hospitals. Box plot of the CTDI_vol_-median value, interquartile range, total range and outliers for all CT-thorax examinations on the five CT systems with the national P75 and P25 DRLs as benchmark

**Fig. 5:**
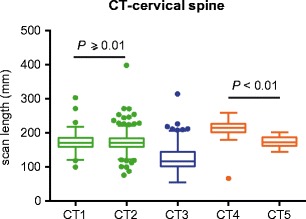
Detection of differences in the scan length of CT-cervical spine studies between the scanners in hospital C. Box plot of the scan length used for CT of the cervical spine on the five CT systems. Adjusted *p* values compare CT1 versus CT2 and CT4 versus CT5, Kruskal-Wallis test with post-hoc Dunn test. Adjusted *p* values not shown on the graph are below 0.01

Dose-tracking systems help to track and achieve dose reduction below the national P75 DRLs, which was the case in hospital B for CT-head and CT-abdomen-pelvis examinations. In hospital B a dose reduction of respectively 27.9% and 33.1% was found when the CTDI_vol_ values of all examinations were compared in a period three months before and three months after the scan parameters were adapted (CT-head: *n* = 1406 and CT-abdomen-pelvis: *n* = 764 examinations evaluated) (Fig. 1a, Fig. E1). Overall, both the CTDI_vol_ (Table 4) and DLP from all scanners for all CT regions were below the national P75 DRLs (Figs. 1, 2, 3, 4 and 5, Fig. E2). Moreover for CT-head (Fig. 3a), CT-lumbar spine (Fig. 1c) and CT-cervical spine (Fig. 1b) studies the median CTDI_vol_ values of all but one scanners were even below or at the 25th percentile (P25) levels of the national CT-dose survey and this was also the case for CT-thorax (Fig. 4) for three of the five scanners.

**Table 4:** Median CTDI_vol_ (mGy) per scanner for the different CT regions. P75 and P25 DRL levels of the nationwide survey as reference. *Separate protocols for the thorax and abdomen with different CTDI_vol_

	CT- head	CT-abdomen-pelvis	CT- thorax	CT-pulmonary embolism	CT-lumbar spine	CT-thorax-abdomen	CT-cervical spine
CT1	25.2	10.8	5.3	12.4		12.5	11.1
CT2	25.6	9.6	6.6	10.3	17.5	12.3	11.4
CT3	53.6	9.0	4.2	11.0	28.6	6.4	30.5
CT4	20.5	4.3	3.0	2.3	12.1		6.1
CT5	18.4	4.0	3.1		13.7	*	6.6
DRL P75	58.0	13.0	10.0	20.0	32.0	13.0	32.0
DRL P25	38.0	7.0	5.0	6.5	17.5	6.5	15.0

Next, the dose data from the two GE CT systems installed in hospital A were compared and the same was done for the two Siemens CT systems installed in hospital C. Early after implementing the dose-tracking system in hospital A, a difference of 60.7% between the CTDI_vol_ levels of CT-head examinations between both identical scanners was observed (median CTDI_vol_ of 23.8 mGy versus 39.2 mGy, *P* value < 0.0001). After aligning protocol parameters, CTDI_vol_ levels were only 1.6% different (median CTDI_vol_ of 23.4 mGy versus 23.8 mGy, *P* value = 0.005) (Fig. 2a).

Overall, both CTDI_vol_ and DLP levels were not significantly different for the two CT systems used in both hospital A and C for the following CT regions: CT-abdomen-pelvis and CT-thorax-abdomen (Figs. 1, 2 and 3, E3 and Table 5). This was also the case for CT-head, but not for CT-pulmonary embolism or CT-thorax examinations in hospital A. In hospital C the DLP levels of CT-head examinations were consistent (Fig. E3, Table 5), but this was not the case for CTDI_vol_ levels (Fig. 3a, Table 5) between their two CT systems. For CT-thorax examinations in hospital C it was the other way around (Fig. 4, Fig. E3, Table 5). For CT-lumbar spine examinations a significant difference in both CTDI_vol_ (Fig. 1c, Table 5) and DLP (Fig. E3, Table 5) between the two CT systems was observed in hospital C. All CT-lumbar spine examinations in hospital A were performed on one CT system and hence intrahospital comparison or “internal benchmarking” was not possible.

**Table 5:** Multiple comparison adjusted *p* values for comparison of median CTDI_vol_ and DLP values between scanners highlighting the intrahospital benchmarking

	CT1 versus CT2		CT4 versus CT5	
	CTDI	DLP	CTDI	DLP
CT-head	> 0.99	> 0.99	< 0.001	> 0.99
CT-abdomen-pelvis	> 0.99	0.04	> 0.99	> 0.99
CT-thorax	0.0001	< 0.0001	0.48	0.001
CT-lumbar spine	NA	NA	< 0.01	< 0.01
CT-pulmonary embolism	< 0.0001	0.03	NA	NA
CT-cervical spine	> 0.99	> 0.99	0.08	> 0.99
CT-thorax-abdomen	0.85	> 0.99	NA	NA

The distribution of the used radiation doses for the same CT regions performed in different hospitals is shown in Figs. 1, 2, 3, 4 and 5 (and Fig. E3). Significant differences between hospitals (median, interquartile range, total range) in both CTDI_vol_ and DLP were observed for all examination types. The differences in median CTDI_vol_ (Table 4) were most obvious for CT-pulmonary embolism (10.3–12.4 mGy in hospital A, 11.0 mGy in hospital B and 2.3 mGy in hospital C) (Fig. 3b), for CT-cervical spine (11.1–11.4 mGy in hospital A, 30.5 mGy in hospital B and 6.1–6.6 mGy in hospital C) (Fig. 1b) and CT-head examinations (25.2–25.6 mGy in hospital A, 53.6 mGy in hospital B, 18.4–20.5 mGy in hospital C) (Fig. 3a), with a 5.4-, 5.0- and 2.9-fold difference between the CT system with the highest and lowest median CTDI_vol_ for CT-pulmonary embolism, CT-cervical spine and CT-head respectively. Hospital C consistently had the lowest median CTDI_vol_ and DLP for all examination types (*p* values < 0.01). Depending on the scanned CT region sometimes hospital A and sometimes hospital B had the second lowest median CTDI_vol_ and DLP.

Hospital C also had the narrowest range of dose values for all CT regions (Figs. 1, 2, 3, 4 and 5). Hospital B (CT 3) had a large range of dose levels for CT-thorax studies, explained by the use of a large number of protocols for many different clinical indications (Fig. 4).

The scan lengths for CT-head, CT-thorax, CT-thorax-abdomen and CT-cervical spine examinations were consistent between the two CT systems in hospital A (*p* values ≥ 0.01) (Fig. 5, Fig. E4), while in hospital C the scan lengths were not consistent (*p* values < 0.01) between their two CT systems for CT-thorax, CT-lumbar spine, CT-cervical spine and CT-head examinations (Fig. 5, Fig. E4). Overall scan lengths per CT region were significantly different between the hospitals, whereby the hospital with the overall lowest dose, hospital C, had the longest scan length (Fig. 5 and Fig. E4).

## Discussion

In this multicentre study we compared dose levels and scan lengths of standard adult CT examinations with similar clinical indications between and within three regionally affiliated institutions, and to national DRLs, using a dose-tracking system. The results of this study illustrate that dose-tracking systems are helpful in benchmarking and in the process of dose optimisation for several reasons.

Recording of the radiation dose (CTDI_vol_, DLP) for every CT study by a dose-tracking system allowed the three participating hospitals to know whether they were using doses below or above the P75 of the national DRLs. When needed, dose optimisation to obtain CTDI_vol_ and DLP values below the P75 was undertaken (adapting mAs, kVp and use of iterative reconstruction). Median dose levels for all procedures were below the P75 and in the smaller hospital even below the P25 level. In this hospital, a long history of CT-dose optimisation existed and it was equipped with the most recent CT scanners (2012). With over 20 years of experience, the lead CT radiologist was confident images were of diagnostic quality, even using these low CT dose values. Regular investment in new CT technology seems to be a good and sometimes necessary policy to achieve further radiation dose optimisation and optimal patient care. If outdated CT equipment is used, further dose optimisation is no longer possible because of technology limitations.

Within the same hospital, the radiation dose used on several and often different CT systems can readily be compared by means of a dose-tracking system. Large differences in radiation dose for comparable clinical indications and patient groups (size and age) between the CT systems are ethically not acceptable and will be detected by dose-tracking software. This triggers an ‘internal’ benchmarking; the radiologists are challenged to optimise the radiation dose on the system with the highest dose until it approaches the dose of the other system, taking into account possible differences in technologies. The hospitals in this study using two CT systems ended up with consistent dose levels after several months of dose-tracking system utilisation.

CT-dose levels between scanners of different hospitals varied significantly for similar CT region studies. This data comparison and exchange between hospitals could trigger hospitals, using higher radiation doses for certain CT studies, to reduce their dose levels or to invest in new dose reduction CT technology. The lowest dose levels for all examinations were found in hospital C where, on top of the newest CT equipment, a single radiologist interested in dose optimisation was responsible for the CT organisation and protocols. This resulted in a limited number of standardised CT study settings and low median dose levels. In the larger hospitals A and B multiple radiologists used their preferred CT study settings with often high variability in radiation dose, resulting in higher median CTDI_vol_ and DLP values. However, when comparing across hospitals, we found that within one hospital dose levels can be in the high or low range depending on the CT region studied. A part of this effect could probably be attributed to a variation in patient populations between the hospitals. For example, hospital B has, compared to hospitals A and C, a larger group of CT-thorax patients in follow-up, requiring lower CT dose levels. While the median CTDI_vol_ of CT-thorax in hospital B was very low, the median CTDI_vol_ of CT-head was by far the highest. The higher levels of CTDI_vol_ of CT-head examinations could not be explained by a difference in patient population as those CT examinations in the three participating hospitals are primarily performed for stroke patients, trauma patients and patients with acute headache, all screening examinations requiring an equal dose, and none of the hospitals performed CT perfusion studies, which do require higher CT dose levels. The reason for the low CTDI_vol_ of CT-thorax in hospital B was that the radiologist most involved in CT was sub-specialised in the thorax, which allowed “CT-thorax setting” optimisation in a stratified way, depending on patient habitus. In the same hospital, attempts to further reduce the dose of the CT-head studies were not successful because the (neuro)radiologists no longer accepted the image quality. Benchmarking will help such a radiology department in finding an acceptable balance between dose and image quality. An important role could be played here by recognised (inter)national organisations, which could provide DRLs tailored to the size, type of pathology, equipment and location of the hospitals.

Using multiple scan protocols for different clinical indications and patient groups (e.g. obese patients) for the same CT region will disperse the CT-dose levels. This was for example the case in hospital B where the CTDI_vol_ of CT-thorax examinations varied substantially, but the median CTDI_vol_ was only slightly higher than in hospital C. Only subtle differences in CTDI_vol_ for the CT-thorax studies were registered in hospital C with the overall lowest median CTDI_vol_. However tailoring ‘scan protocols’ allows administering higher or lower CT doses to the right patients (higher in obese patients, lower in young and slim patients, etc.), resulting in optimal ‘dose-image quality’ while maintaining a low median CTDI_vol_. Dose monitoring and benchmarking can warn radiologists, who are using multiple scan protocols, when the median CTDI_vol_ is too high or can inform those using a restricted number of scan protocols when patients could benefit from a more tailored lower dose protocol. Being a topic of future research, grouping subjects/procedures according to clinical indication will most likely reduce the heterogeneity of the data and increase the interpretability of results.

In one hospital a difference in scan length was observed between their two CT systems in the majority of the CT examination types. On the technologically most advanced system the scan length was automatically set on the topogram by the vendor’s software. This resulted in an unnecessary long scan length compared to the scan length set manually on the other scanner. Dose-tracking systems and internal/external benchmarking will detect these differences and can therefore help to correct suboptimal vendor protocol settings, resulting in further dose optimisation.

One of the limitations of this study was that only CT examinations with one helical acquisition were compared. Complex CT studies with multiple helical acquisitions were not compared as their data collection and comparison are more difficult, and currently our government does not yet collect these data to establish national DRLs. The purpose was also to include CT studies performed for exactly the same or comparable clinical indications. However, this could not always be achieved and the choice to include one regional hospital, one large city hospital and one university hospital itself led to inclusion of patients with different kinds of pathology. Although the CT studies in this work comprise the majority of the daily CT load, many other CT studies (multiphase studies, polytrauma, coronary angiography, biopsy guided CT procedures, etc.) were not evaluated.

In 2014 the European Union published a new directive regarding safety matters in radiation protection [25]. The directive states that recording and reporting the radiation level in every patient and in every medical practice will be required in the EU and must be implemented in January 2018. It is clear that dose-tracking systems will play a key role in the management of the big data stream of this future obligation. Large data collection allows gathering information of a more representative population that can be used to establish national DRLs. In conclusion dose-tracking systems allow comparing and sharing dose, scan length and protocol data between and within hospitals and have the potential to further reduce dose variability of standard adult studies, even when these are already below national DRLs.

## Electronic supplementary material

ESM 1 (DOCX 284 kb)

## Abbreviations

CT: Computed tomography
CTDI_vol_: Volume computed tomography dose index
DLP: Dose length product
DRL: Dose reference level
P75: 75th percentile
P25: 25th percentile
