# Supplementary material for: Benchmarking adult CT-dose levels to regional and national references using a dose-tracking software: a multicentre experience
Source: Insights Imaging. 2017 Sep 7;8(5):513–21. doi: 10.1007/s13244-017-0570-5 (PMC5621994; doi:10.1007/s13244-017-0570-5)

**Supplemental Material**

**Supplemental figures**

**Figure E1:** Graph showing the median CTDI_vol_ and interquartile range per month (period February 2013 - August 2013) of CT-abdomen-pelvis examinations in hospital B with the national P75 DRL as benchmark


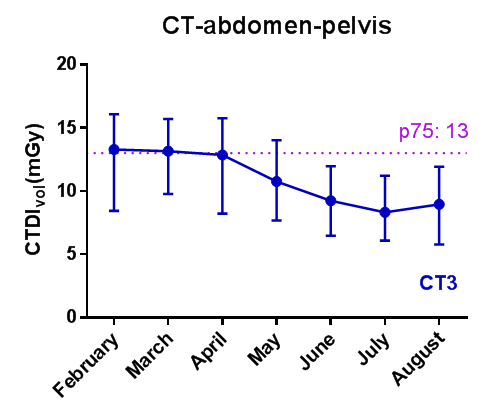


**Figure E2:** Median CTDI_vol_ and DLP per hospital and per CT-region with the national P75 and P25 DRLs as benchmark levels.


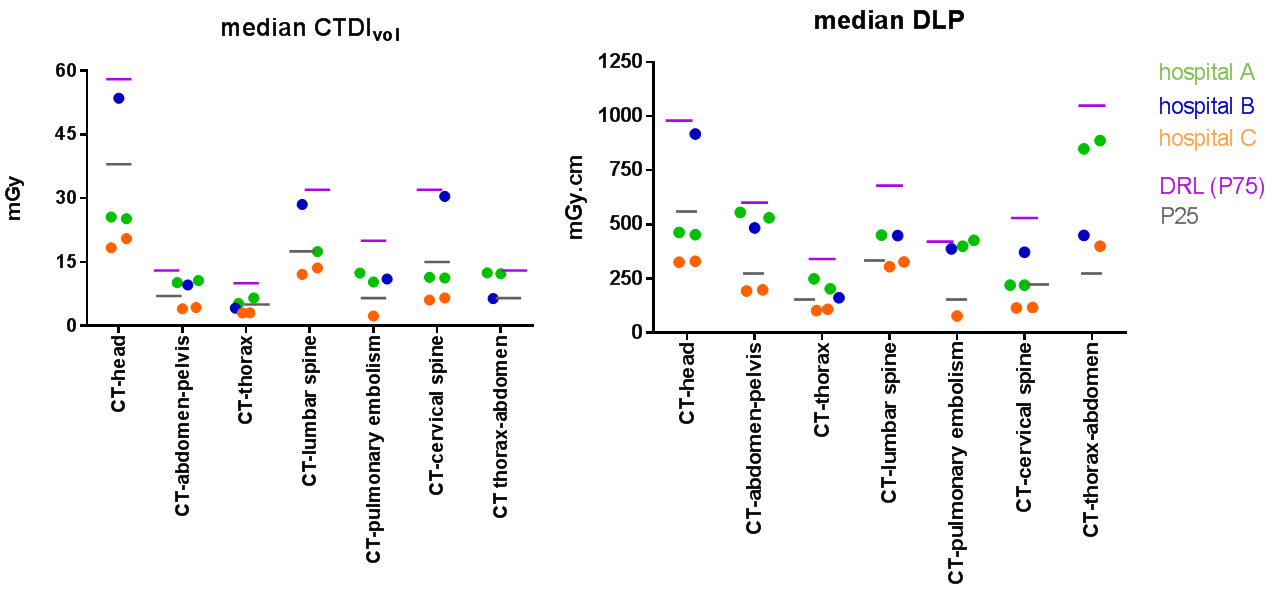


**Figure E3:** Box plot of the DLP - median value, interquartile range, total range and outliers for all CT-head, abdomen-pelvis, thorax, lumbar spine, cervical spine, thorax-abdomen and pulmonary embolism examinations on all CT-systems with the national P75 and P25 DRLs as benchmark.


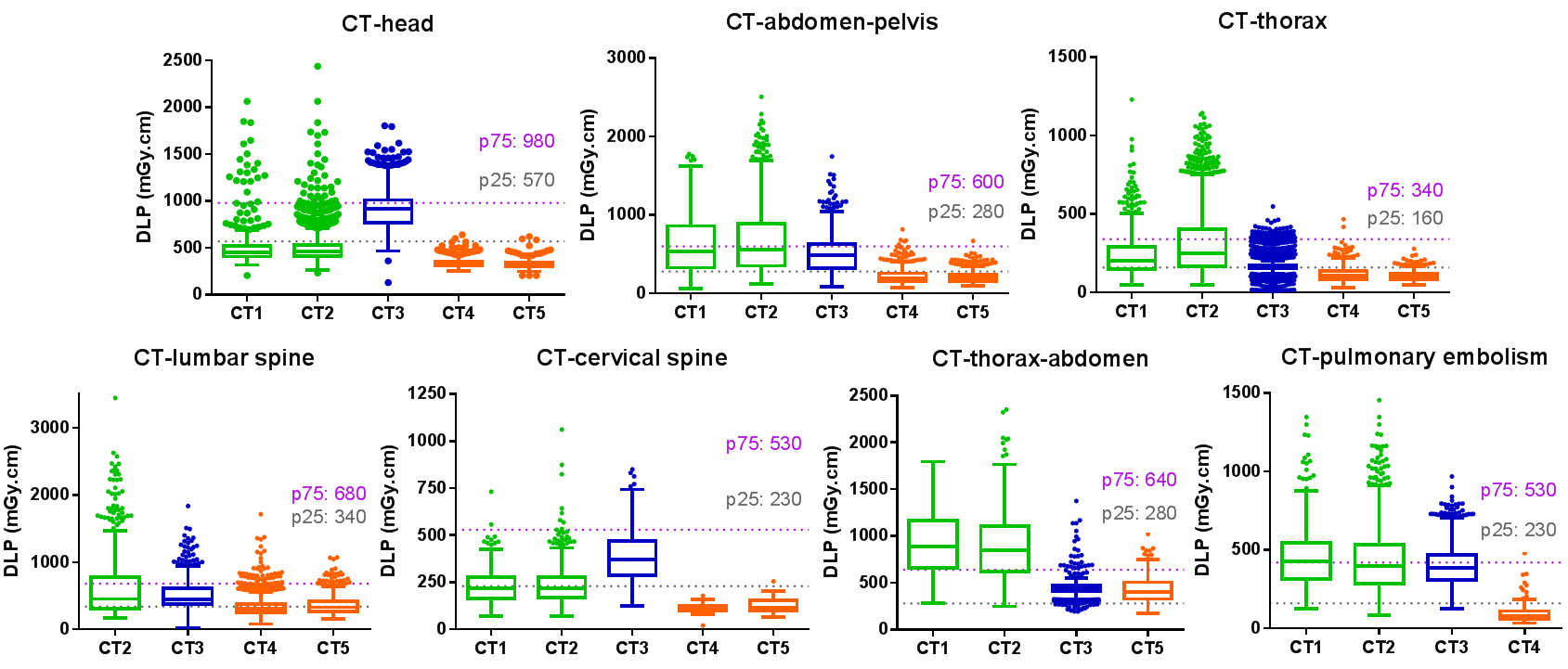


**Figure E4:** Box plot of the scan length used for CT-head, abdomen-pelvis, thorax, lumbar spine, pulmonary embolism and thorax-abdomen on all CT-systems


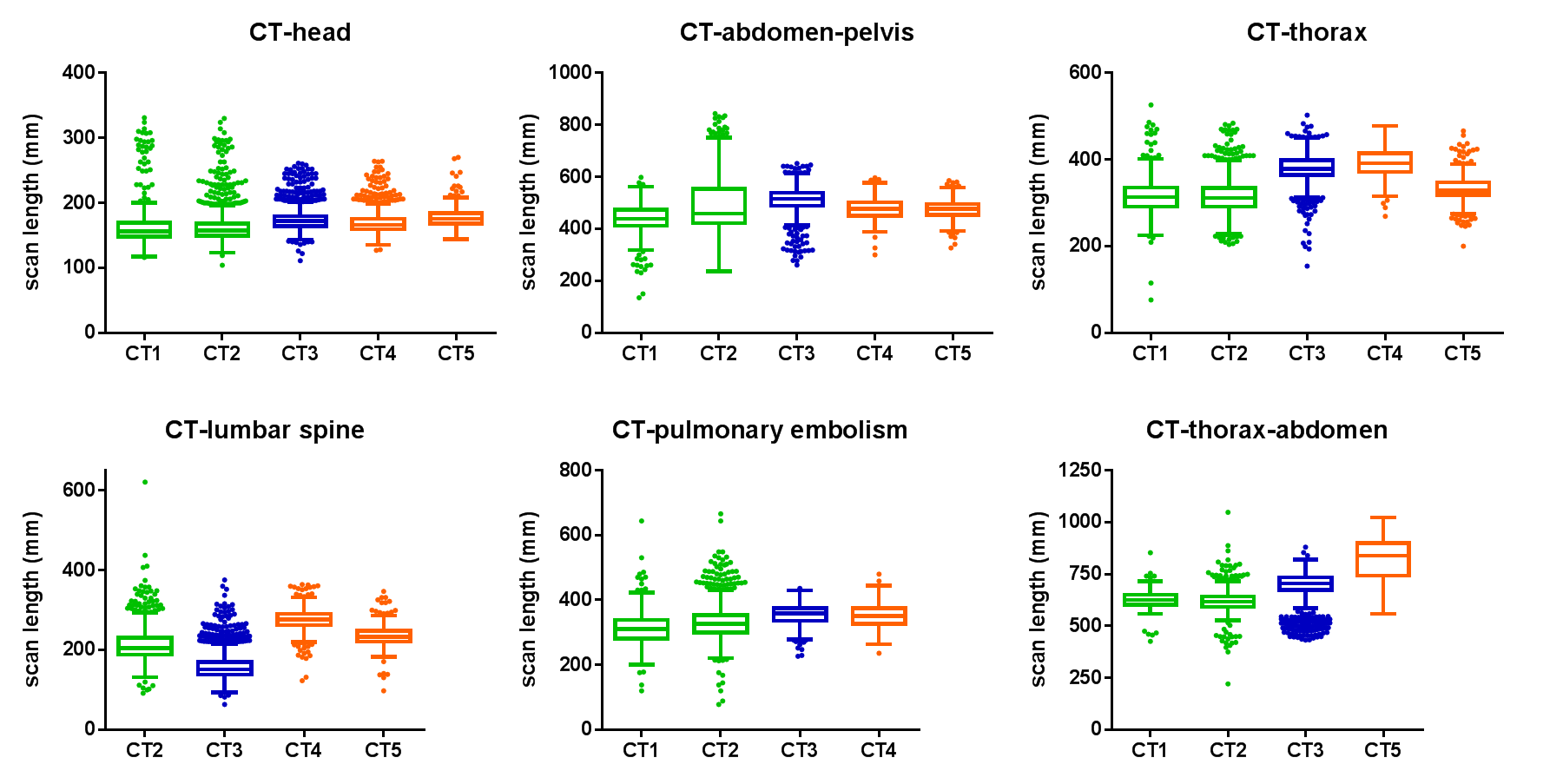

Supplement: Supplementary file 1 — (DOCX 284 kb) [file 13244_2017_570_MOESM1_ESM.docx]
